# Model driven optimization of antiangiogenics + cytotoxics combination: application to breast cancer mice treated with bevacizumab + paclitaxel doublet leads to reduced tumor growth and fewer metastasis

**DOI:** 10.18632/oncotarget.15484

**Published:** 2017-02-18

**Authors:** Severine Mollard, Joseph Ciccolini, Diane-Charlotte Imbs, Raouf El Cheikh, Dominique Barbolosi, Sebastien Benzekry

**Affiliations:** ^1^ SMARTc Unit, Inserm S_911 CRO2, Aix Marseille University, Marseille, France; ^2^ Cancer Research UK Cambridge Institute, University of Cambridge, Cambridge, UK; ^3^ MONC Team, INRIA, Bordeaux, France

**Keywords:** tumor vasculature normalization, antiangiogenics, scheduling, combination, mathematical modeling

## Abstract

Bevacizumab is the first-in-class antiangiogenic drug and is almost always administrated in combination with cytotoxics. Reports have shown that bevacizumab could induce a transient phase of vascular normalization, thus ensuring a better drug delivery when cytotoxics administration is adjuvant. However, determining the best sequence remains challenging. We have developed a mathematical model describing the impact of antiangiogenics on tumor vasculature. A 3.4 days gap between bevacizumab and paclitaxel was first proposed by our model. To test its relevance, 84 mice were orthotopically xenografted with human MDA-231^Luc+^ refractory breast cancer cells. Two sets of experiments were performed, based upon different bevacizumab dosing (10 or 20 mg/kg) and inter-cycle intervals (7 or 10 days), comprising several combinations with paclitaxel. Results showed that scheduling bevacizumab 3 days before paclitaxel improved antitumor efficacy (48% reduction in tumor size compared with concomitant dosing, *p* < 0.05) and reduced metastatic spreading. Additionally, bevacizumab alone could lead to more aggressive metastatic disease with shorter survival in animals. Our model was able to fit the experimental data and provided insights on the underlying dynamics of the vasculature's ability to deliver the cytotoxic agent. Final simulations suggested a new, data-informed optimal gap of 2.2 days. Our experimental data suggest that current concomitant dosing between bevacizumab and paclitaxel could be a sub-optimal strategy at bedside. In addition, this proof of concept study suggests that mathematical modelling could help to identify the optimal interval among a variety of possible alternate treatment modalities, thus refining the way experimental or clinical studies are conducted.

## INTRODUCTION

Launched in 2004, bevacizumab has been approved since then in a variety of settings in solid tumors such as colorectal, breast, lung or ovarian cancers, with mixed and sometimes still questioned impact on survival [1]. Of note, bevacizumab has always only been approved as a concomitant administration with associated cytotoxics. Several studies from independent academic groups have suggested that anti-angiogenics could induce a transient phase or vasculature normalization with increased tumor blood perfusion, prior to exerting its antiangiogenics properties [2–6]. This paradoxical action has been considered as possibly generating a time-window to administrate chemotherapeutic agents, thus suggesting a paradigm shift from concomitant to sequential dosing. Indeed, delaying chemotherapy could allow higher quantities of drugs to reach the tumor, provided that their administration coincides with this normalization phase. As early as 2004, it has been shown that blocking VEGFR2 could decrease tumor hypoxia at the beginning of the treatment, thus demonstrating that transient normalization of tumor neo-vessels happens indeed with antiangiogenics [7, 8]. This was already associated with improved efficacy of combined radiotherapy or chemotherapy. Indeed, because disrupted tumor vasculature may lead to resistance to chemotherapy and radiotherapy due to subsequent higher interstitial fluid pressure, and reduced blood flow lowering drug delivery [9], alternate scheduling with antiangiogenics could overcome these resistances. Ever since, several groups have worked on this issue, mostly as part of experimental therapeutics [4, 6, 10]. Only few clinical trials have investigated on alternate scheduling with bevacizumab. The BRANCH study evaluated bevacizumab in rectal cancer patients after standard concomitant dosing or alternative sequential administration. Whereas concomitant dosing was little effective, the sequential administration led to 50% of tumor regression rate with 85% of 5-years survival and better tolerance [11]. These promising results supported the ongoing OBELICS study (Optimization of BEvacizumab scheduLIng within Chemotherapy Scheme), a phase-3 trial that will compare different sequences of bevacizumab associated with chemotherapy [12]. Despite these encouraging findings, the need for identifying proper biomarkers to forecast bevacizumab impact on neovessels remains critical [13] and until they are made available, *in silico* tools could be helpful to optimize alternate schedules. In contrast to the many pharmacodynamic models describing the action of cytotoxics on tumor growth [14], and despite substantial theoretical efforts in the field of cancer modeling to simulate angiogenesis and tumor-vasculature interactions [15–18], relatively few mathematical models of anti-angiogenic therapy have been actually confronted to experimental data [19, 20], and even less have investigated combined effects of cytotoxics with antiangiogenics [20, 21]. To address this issue, our group has developed a phenomenological model describing the effect of antiangiogenics on vasculature quality throughout time, thus potentially forecasting when the normalization phase occurs [22]. When coupled with an efficacy component, this model should allow to compare *in silico* differences in efficacy depending on the lag-time between cytotoxics and antiangiogenics, thus helping in decision-making prior to start the actual experiments. As a proof-of-concept study, the aim of the present work was to confront model simulations with experimental data generated in a canonical refractory breast cancer mice model (i.e., MDA-MB231) treated with a standard combo between anti-angiogenics and cytotoxics (i.e. bevacizumab-paclitaxel doublet) in this setting.

## RESULTS

**Figure 1:**
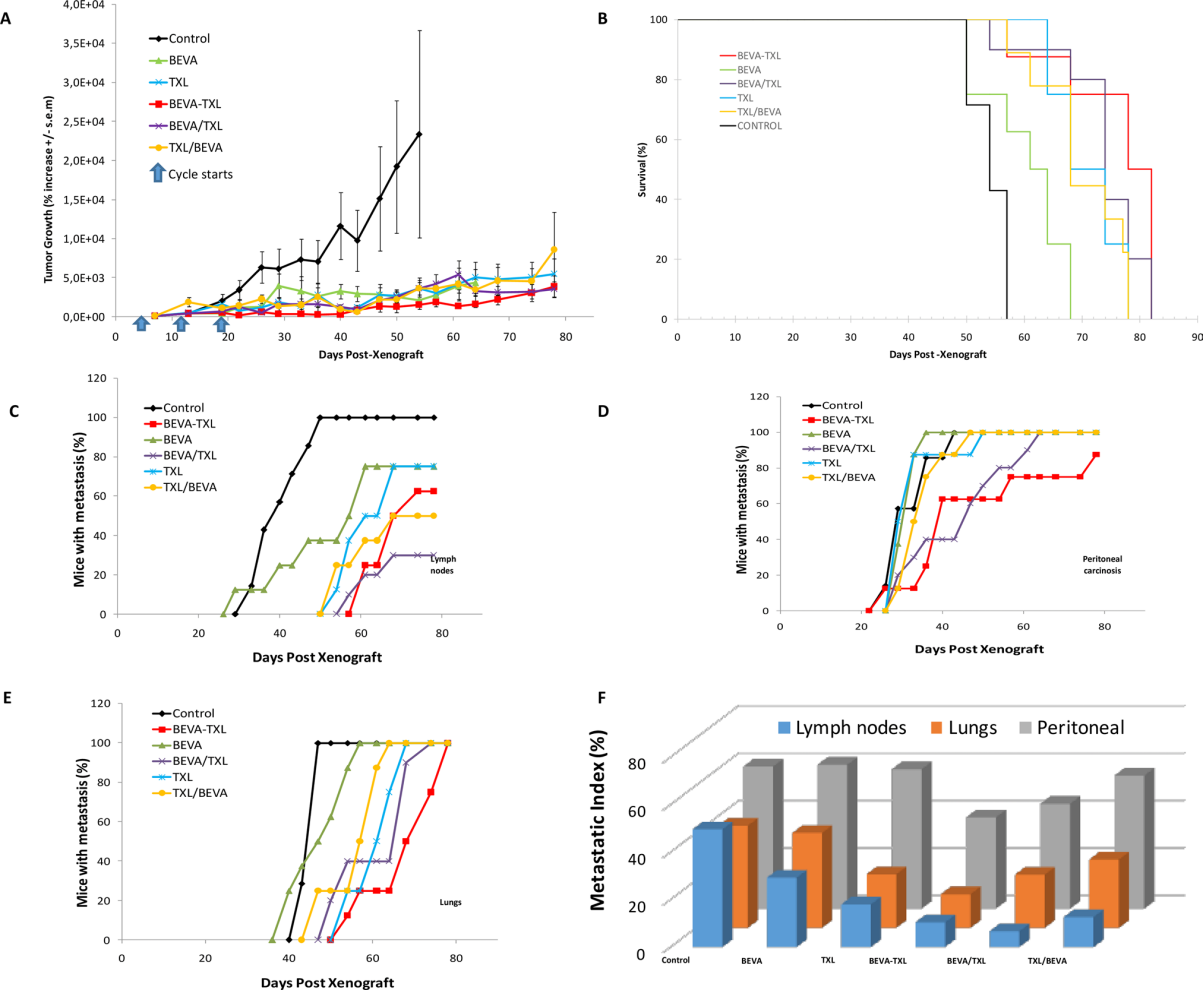
Monitoring tumor growth, survival and metastatic spreading in Expe-1 Bevacizumab and paclitaxel were used at 10 and 20 mg/kg I.P, respectively, with a 3 days lag when used sequentially. Three courses were administrated every 7 days (D5, D12, D19). Treatments were as following. Control: saline, BEVA: single angent bevacizumab, TXL: single agent paclitaxel, BEVA-TXL: bevacizumab and paclitaxel given concomitantly, BEVA/TXL: bevacizumab followed by paclitaxel, TXL/BEVA: paclitaxel followed by bevacizumab. (**A**). Tumor mass was evaluated by 2D bioluminescence imaging and expressed as % increase from initial measurement at D8. Values are mean ± s.e.m. (**B**). Survival. Mice were sacrificed when tumor volume reached an apparent mass of > 2g, or when signs for pain or distress were observed. Mice were imaged at 6 different wavelengths to discriminate different light-emitting sources on several plans. Secondary signals were gathered as 3 different groups: lymph nodes (**C**), peritoneal carcinosis (**D**) and lung metastasis (**E**, **F**). Metastatic indices (areas under the metastatic incidence curves) for the different groups and metastatic locations.

**Figure 2:**
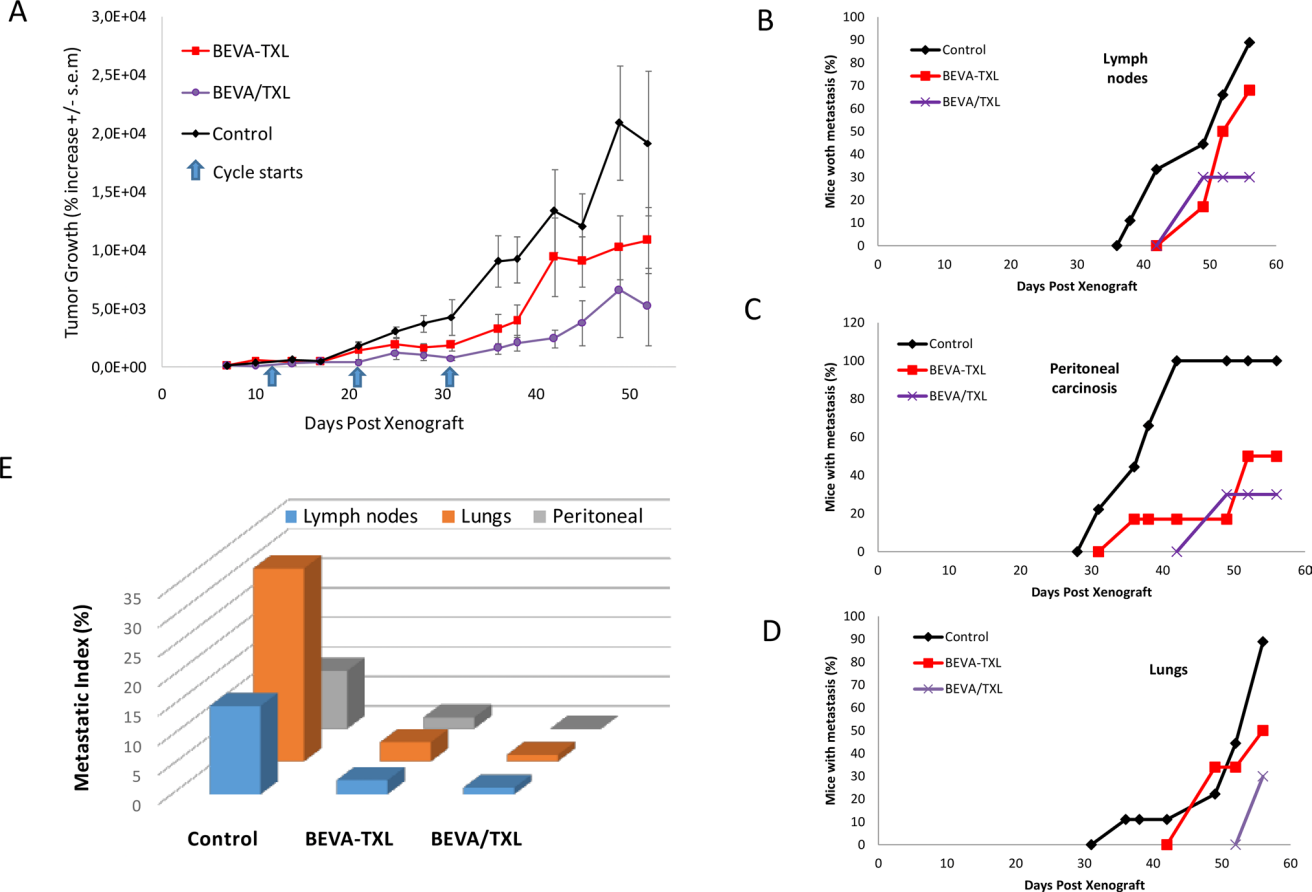
Monitoring tumor growth and metastatic spreading in Expe-2 Both bevacizumab and paclitaxel were used at 20 mg/kg I.P., respectively, with a 3 days delay when used sequentially. Three courses were administrated every 10 days (D11, D21, D31). Treatments were as following: Control: saline, BEVA-TXL: bevacizumab and paclitaxel given concomitantly, BEVA/TXL: bevacizumab followed by paclitaxel. (**A**) Tumor mass was evaluated by 2D bioluminescence imaging and expressed as % increase from initial measurement at D8. Values are mean ± s.e.m. Metastatic lesions were screened and quantitated by 3D bioluminescence imaging with DLIT reconstruction. (**B**): lymph node metastasis, (**C**): perinotenal carcinosis, (**D**): lung metastasis. (**E**). Metastatic indices Metastatic indices (areas under the metastatic incidence curves) for the different groups and metastatic locations.

Based on our previous theoretical work [22] we performed simulations to inform the optimal time lag between the administrations of the two drugs (Supplementary Figures 1 and 2). These suggested an optimal gap of 3.4 days, leading to a putative reduction of 15.4% of the tumor mass as compared to a concomitant administration. Although based on parameter values that were not obtained from a quantitative fit to experimental data, we used this value as a starting point and fixed a time lag of 3 days in the BEVA/TXL group of Experiment-1. In this experiment, no difference was observed in carcass weight among the different treatment groups (data not shown). Monitoring of tumor growth is shown in Figure 1A. At the end of the treatment phase (one week after the third cycle, i.e. D26), mean tumor mass expressed as % of initial mass were 6339 ± 1999 (Control), 1270 ± 470 (Beva), 1222 ± 372 (TXL), 626 ± 234 (BEVA-TXL), 549 ± 245 (BEVA/TXL) and 2260 ± 553 (TXL/BEVA). A statistical difference was found between the groups (*p* = 0.007, Anova on the Ranks). Further Dunn's multiple comparison testing showed that all treatment groups but BEVA and TXL/BEVA were different than Control (*p* < 0.05). At study conclusion (D78), mean tumor sizes (% of the initial mass) in the 4 remaining groups were 5460 ± 2000 (TXL), 3857 ± 1370 (BEVA-TXL), 3560 ± 970 (BEVA/TXL) and 8585 ± 4860 (TXL/BEVA). However, the differences between the remaining groups at study conclusion were not statistically significant (*p* = 0.788, Anova on Ranks). Survival curves for Experiment-1 are displayed in Figure 1B. Median survivals were 54 days (Control), 60 days (BEVA), 68 days (TXL), 68 days (TXL/BEVA), 74 days (BEVA/TXL) and 78 days (BEVA-TXL). A statistical difference in survival was found between the groups (*p* = 2.4 10^−7^), but further log rank testing showed that survival in the BEVA group was not different than Control (*p* = 0.0898), and no significant difference was evidenced between the BEVA/TXL and the BEVA-TXL groups (*p* = 0.214). Metastatic lesions were monitored and measured using 3D imaging (Supplementary Figure 3). Three main metastatic sites were found: lymph nodes, peritoneal carcinosis, and lungs. All the animals had at least one metastatic site. The kinetics of metastasis apparition throughout time is displayed in Figure 2C–2E. Marked differences were observed between the groups and the metastatic sites, both in terms of time and number of animals presenting with metastasis. In the Control group, 100% of the mice developed metastasis and all animals developed secondary lesions on the 3 different sites. Time to reach 50% animals with at least one metastatic lesion were 29 days (Control), 33 days (BEVA, TXL, and TXL/BEVA), 40 days (BEVA-TXL) and 47 days (BEVA/TXL). Metastasis-free survival analysis (log-rank) revealed a significant difference between all groups (*p* = 9.13 × 10^−11^, 0.0031 and 1.73 × 10^−9^ for the axillary, peritoneal and lung locations, respectively). However, significant differences between the BEVA/TXL and BEVA-TXL groups were not obtained for any site. Nevertheless, only 30% of the mice had axillary node invasion at study conclusion in the BEVA/TXL group versus 62.5% in the BEVA-TXL group. To further quantify the metastatic dynamics, a metastatic index was defined for each group and each location as the area under the cumulative incidence divided by the duration of the experiment (78 days in Experiment-1). This new metrics thus represents the average fraction of mice with metastasis over the experiment's duration and is reported in Figure 1F.

**Figure 3:**
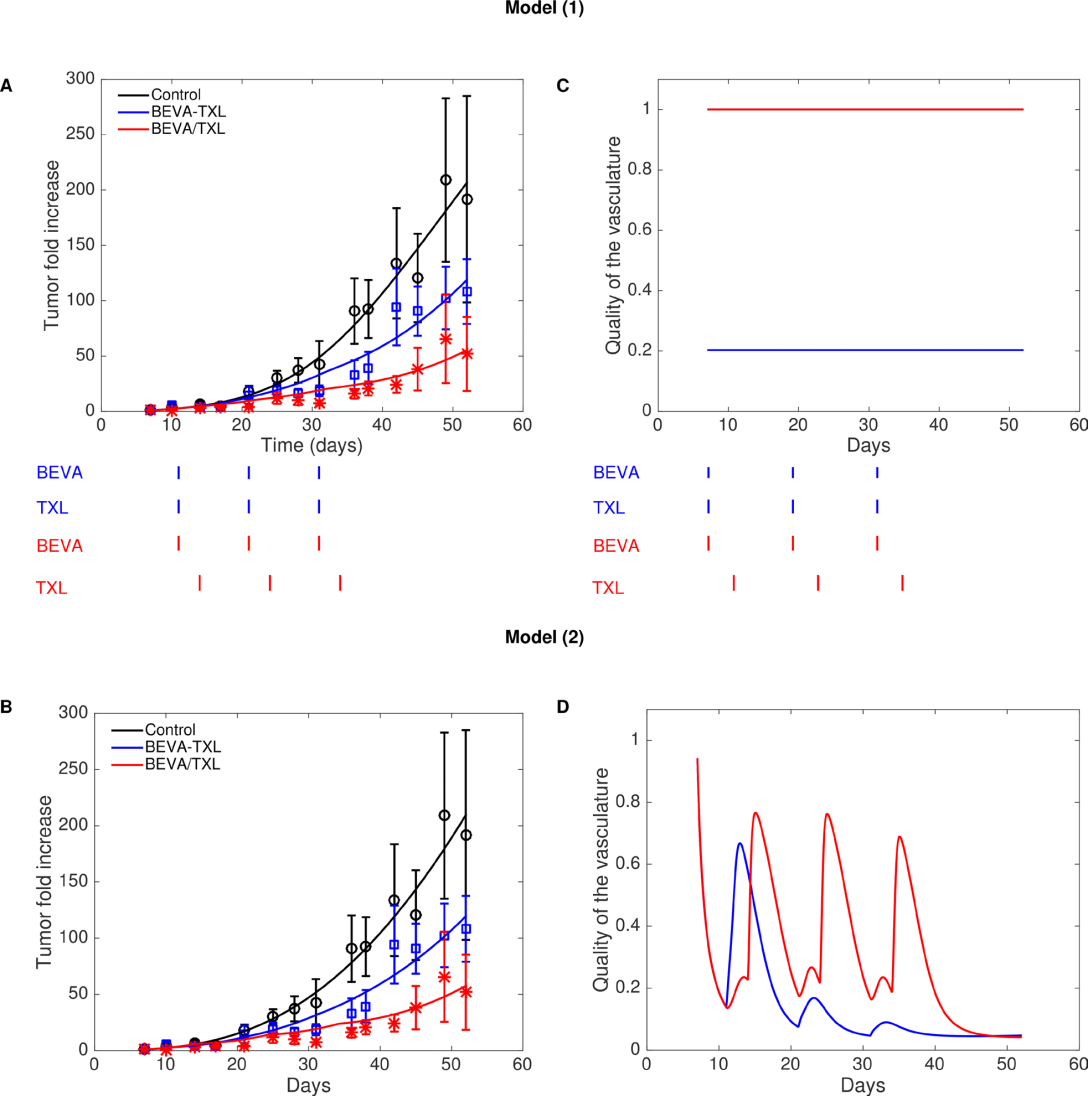
Fits of models (1) (**A**) and (2) (**B**) to Expe-2 and resulting inferred quality of the vasculature (**C**) and (**D**).

For Experiment-2, Figure 2A shows mean tumor growth among the groups expressed as % of initial mass. At the end of the treatment phase (one week after the third cycle, i.e. day 38), a significant difference was found between the groups (*p* = 0.032, Anova on the Ranks). Further Dunn's multiple comparison testing showed that BEVA/TXL group, but not BEVA-TXL group, was different than Control (*p* < 0.05). At study conclusion (D52), mean tumor sizes (% of the initial mass) were 19173 ± 9325 (Control), 10832 ± 2929 (BEVA-TXL), and 5186 ± 3341 (BEVA/TXL). A significant difference was found again between the groups (*p* = 0.012, Anova on the Ranks). Further Dunn's multiple comparison testing showed that BEVA/TXL group, but not BEVA-TXL group, was different than Control (*p* < 0.05). The kinetic of metastasis apparition throughout time depending on the localization and the treatment group is displayed in Figure 3B–3D. At study conclusion, in the Control and BEVA-TXL groups, 100% and 68% of the mice developed at least one metastasis whereas only 30% of the animals in the BEVA/TXL group displayed a metastasis. Time to reach 50% animals with at least one metastatic lesion was 38 days (Control), 52 days (BEVA-TXL) and was not reached by study conclusion for BEVA/TXL. Regarding the metastatic index, the BEVA/TXL group exhibited smaller values than BEVA-TXL for all locations (Figure 2E). The animals were necropsied at D60 to compare macroscopic search for metastatic lesions and 3D imaging. All the lesions previously identified by bioluminescence were confirmed upon autopsy (data not shown).

**Figure 4:**
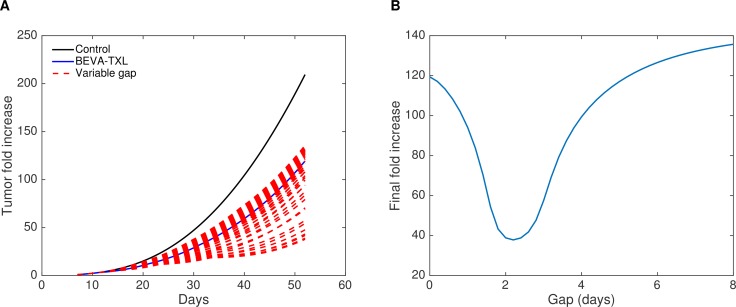
Data-informed modeling simulations of various gaps between bevacizumab and paclitaxel administrations The interval (gap) ranged from 0 (concomitant) to 8 days. (**A**) Tumor growth kinetics. (**B**) Final tumor fold increase as a function of the gap.

Our two mathematical models were able to fit the data of Experiment-2, with model best-fits falling within error bars for most of the measurements (Figure 3A and 3B). Resulting parameter values are reported in the Supplementary Table 1. Note that the growth and treatment parameters were kept the same among the groups and only the scheduling of the drugs was changed. The fit of the simple model (1) allowed to quantitatively estimate improvement of vasculature quality due to bevacizumab and yielded a value of *Q–* of 0.203 (Figure 3C), suggesting a 5-fold increase in drug delivery when bevacizumab was administered first. Figure 3B shows further modeling using model (2) with dynamic and semi-mechanistic evolution of the quality of the vasculature depending on the vascular variables of the model. This last model allowed inference of unobserved quantities (such as *U* and *S*), generating insights on the dynamics of the system (Supplementary Figure 4). The dynamics of the quality of the vasculature *Q* revealed interesting patterns (Figure 3D). First, it exhibited an initial drop from a baseline value of 1 to a low quality, consistently with the reported poor quality of the tumor vasculature when untreated [8, 23–25]. Then, following the first injection of bevacizumab, the number of unstable vessels *U* dropped (Supplementary Figure 4) while keeping a relatively constant level of stable vasculature *S*, resulting in an increase of the quality $Q=\frac{S}{S+U}$ Interestingly, an unexpected effect occurred in the simulation, where two phases of vasculature improvement were observed in the BEVA/TXL schedule (Figure 3D). The first peak corresponds to the action of the bevacizumab alone on the reduction of *U*. The second and larger peak occurred approximately at the time of the cytotoxic injection. It can be explained as follows: the important decrease in *V* after the cytotoxics administration induced a loss in the stimulation term of the equation on *U*. The decrease was more pronounced in the stimulation term than in the inhibition term due to the 2/3 power in the latter. This translated into an important drop in *U* and therefore an increase of *Q* (Supplementary Figure 4 and Figure 3D). Eventually, we could generate *a posteriori* data-informed estimates of the best time window for the post-bevacizumab administration of paclitaxel (Figure 4). This gave a value of 2.2 days as the optimal gap between administrations of the two drugs, yielding a 68.3% tumor size reduction at day 52 when compared to the concomitant schedule, representing an additional 16.5 points in size reduction. Interestingly, the model also predicted that some sequential schedules could be detrimental and provoke an increase when compared with the concomitant schedule, such as an 8 days gap (+13.6%).

## DISCUSSION

Attempts to determine a better way to use bevacizumab are probably as old as the drug itself [26] and several non-clinical models have suggested that although empirical, alternate scheduling would perform better than standard concomitant dosing [4, 5, 8, 11, 26]. Recently, clinical data have shown that bevacizumab combined with carboplatin/nab-paclitaxel doublet yielded higher survival in patients with improved vascularization, probably through increased drug delivery of the nanoparticles [27]. Defining better sequencing between bevacizumab and cytotoxics can be done experimentally, i.e. by monitoring changes in vascular density, hypoxia or tumor blood flow to determine next when the cytotoxics should be given, or in a merely empirical fashion, by testing and comparing different sequences in a trial-and-error mode. In this context, computational approaches could help, through *in silico* modeling, to determine rapidly the best scheduling among countless possibilities. For robustness purposes, and since our data was composed of macroscopic tumor growth kinetics, our mathematical model was deliberately kept to a minimal number of parameters and specifically designed for capturing normalization dynamics following the administration of bevacizumab. Both cytotoxic and anti-angiogenic properties of paclitaxel were integrated in our model (27). However, for the sake of practical usability and identifiability of the model, we skipped the thorough mechanistic description of the process as proposed by other models [15–18], or more detailed modeling of the anti-VEGF effect of bevacizumab [28, 29] or paclitaxel tumor penetration [30]. Despite this, considerable standard errors remained in the estimation of the parameters, in part due to the large uncertainty in the measurement themselves, which is intrinsic to the measurement technique (kinetics of luciferin distribution in heterogeneous tumor mass). This generic model was customized to simulate a variety of sequences between bevacizumab and paclitaxel. We chose this doublet because it is the first-line treatment for HER2-negative breast cancer extensively used in the clinics for a decade now [31]. Similarly, MDA-MB-231 was chosen as a canonical xenograft model for refractory breast cancer. *In silico* simulations first suggested a 3.4 days lag-time when administrating bevacizumab and paclitaxel. For practical reasons, a sharp 3-days sequence was tested experimentally, and bevacizumab was administered I.P. because previous reports have demonstrated that drug distribution and antitumor efficacy after I.P. injection were equivalent to that of I.V. route [32]. In Experiment-1, both the BEVA-TXL and the BEVA/TXL groups displayed significantly reduced tumor growth as compared with all other treatment groups. However, despite a trend towards reduced tumor growth at treatment conclusion (−12%) and study conclusion (−9%), no statistical difference was found in efficacy or survival with the BEVA/TXL sequence over concomitant administration, much probably because of the sub-optimal dosing of bevacizumab in this first experiment (i.e., 10 mg/kg). Of note, the reversed sequence TXL/BEVA showed limited efficacy because no statistical difference was found with Control at treatment conclusion, thus demonstrating that in addition to merely sequencing the dosing, the order of administration does matter indeed. Monitoring metastatic spreading showed marked differences among the groups with sometimes contradictory trends depending on the localization (Figure 2C–2E). Of note, BEVA monotherapy seemed to increase the appearance of both lymph node and lung metastasis, and to a lesser extent of peritoneal carcinosis. This observation that antiangiogenics used alone could trigger early metastatic processes has already been reported with sunitinib in a renal carcinoma mice model [33]. However, this is the first time that it is reported with bevacizumab, and this observation is in line with the significant shorter median survival (60 days) observed with the BEVA group. Based on this first set of data, Experiment-2 was performed with slight changes to achieve a better efficacy with the BEVA/TXL sequence over the BEVA-TXL concomitant group. Bevacizumab dosing was doubled to 20 mg/kg, and interval between two cycles was extended to 10 days. With this new setting, Experiment-2 showed that the BEVA/TXL sequence was more efficient with a statistically significant reduction of 48% and 53% of tumor growth as compared with concomitant dosing at the end of the treatment period and at study conclusion, respectively. Similarly, the BEVA/TXL sequence led to fewer and slightly delayed metastatic lesions, thus confirming the model predictions that delayed administration of paclitaxel achieves better antiproliferative efficacy. Based on the collected data, new model simulations suggested eventually that a 2.2 days delay (i.e., about 53 hours) should be used to administrate paclitaxel. Our model could easily be adapted to other drugs by changing the PK component adequately, which could lead to different optimal windows in other settings. Previous attempts to model combination therapies between antiangiogenics and cytotoxics had first been merely theoretical, i.e. with no quantitative comparison to experimental data [15, 18, 34], apart from two recent studies [20, 21]. Here, our mathematical model was able to fully reproduce experimental data generated in xenografted mice, thus illustrating its robustness and the fact that although not mechanistic, it could mimic the different pharmacodynamic effects of the combination, depending on dosing and scheduling. As such, this model could be used in a prospective way to refine bevacizumab-based combinational regimen, rather than trying to find an optimal sequence in an empirical fashion. Integrating modeling support is now a rising trend not only in basic research, but as well now in experimental and clinical oncology. To date, most resources in computational oncology have focused on developing highly sophisticated mechanistic models to better understand tumor biology. In addition to useful but complex multi-scale approaches, developing simplified PK/PD models with tumor biology kept at its minimal expression to generate configurable parameters is another appealing strategy [14]. Such simplified phenomenological modeling has already proved to be capable of mimicking complicated phenomena [35] and its ability to be implemented at bedside [36–38]. Here, this proof-of-concept study strongly suggests that simplified modeling could help to address the issue of finding the optimal dosing and scheduling with bevacizumab.

## MATERIALS AND METHODS

### Pharmacokinetics (PK) modeling

Plasma concentration of paclitaxel was described by a two-phase profile (absorption and elimination) and was characterized by a relatively fast elimination rate (terminal half-life of 3 hours) [39]. PK parameters of plasma distribution and elimination after intra-peritoneal administration were taken from [39]. The PK of bevacizumab was characterized by a one compartment model with absorption compartment [40]. Supplementary Figure 5 depicts the concentration profiles of the sequential and concomitant administrations for the second experiment and the PK equations. PK parameters are summarized in Supplementary Table 1.

### Pharmacodynamics (PD) modeling

To model the combined action of antiangiogenics and cytotoxics on tumor growth, we departed from the Hahnfeldt model that is able to take into account the effect of vasculature-targeting agents [19]. This model combines Gompertz growth for the tumor volume *V(t)* (with *t* denoting the time) with a dynamic carrying capacity *K(t)* (instead of a constant *K* in the classic Gompertz model). The dynamics of *K(t)* is governed by a balance between endogenous stimulation and inhibition of angiogenesis, with terms derived from biophysical considerations about diffusion rates of stimulatory/inhibitory agents. The effect of the cytotoxic drug (paclitaxel) was modeled similarly as in [41] where the authors considered a delay in the effect the drug, due to the fact that the cells are not directly removed after cytotoxic administration because they only die when reaching a specific step of the cell cycle. After being affected by paclitaxel, the tumor cells thus stop proliferating and go through three compartments *Z*_1_, *Z*_2_ and *Z*_3_ before being removed from the system. Following reported observations, we considered that paclitaxel also had an anti-angiogenic effect [42]. Based on the literature [43], vascular endothelial cells were estimated to be approximately 5 times more sensitive to paclitaxel than tumor cells. The drugs delivery was assumed to be modulated by the vascular capacity, represented in the model by the variable *K(t)*. Concentrations of the cytotoxic and antiangiogenic drugs (respectively denoted *C(t)* and *A(t)*) were given by their respective pharmacokinetics models and parameters. To quantify the effect of vascular normalization following bevacizumab therapy as the key element of this work, we introduced a new variable, the quality of the vasculature, denoted by *Q*. We first considered a model where *Q* is constant in time and depends on the scheduling. The model writes:

(1) $$\{\begin{array}{ll} \frac{dV}{dt}=aV\ln\left(\frac{K}{V}\right)-e_{TXL}KQCV & V\left(t_{I}\right)=1\\ \frac{dK}{dt}=bV-dV^{\frac{2}{3}}K-(e_{BEVA}+5e_{CT})KQAK & K\left(t_{I}\right)=\;K_{0}\\ \frac{dZ_{1}}{dt}=e_{TXL}KQCV-kZ_{1} & Z_{1}\left(t_{I}\right)=0\\ \frac{dZ_{2}}{dt}=kZ_{1}-kZ_{2} & Z_{2}\left(t_{I}\right)=0\\ \frac{dZ_{3}}{dt}=kZ_{2}-kZ_{3} & Z_{3}\left(t_{I}\right)=0\;\\ N=V+Z_{1}+Z_{2}+Z_{3} & N\left(t_{I}\right)=1 \end{array}$$

where *V, K* and Z_*i*_, *i* = 1,…,3 were expressed in normalized units by the initial bioluminescence signal corresponding to the number of injected cells. Thus, *N* represents the fold increase of the total number of cells to their initial value. Initial conditions at the first observation time *t_I_* was thus taken to 1 for *V*. For *K*, it was determined from a preliminary fit to the control data only. Critically, in this first model, the expression of *Q* was given by:

$$Q=\{\begin{array}{cc} \begin{array}{c} \bar{Q}<1\\ 1 \end{array} & \begin{array}{c} \text{for the BEVA}-\text{TXL group}\\ \text{for the BEVA}/\text{TXL group} \end{array} \end{array}$$

where $\bar{Q}$ is a *constant* parameter to be estimated and BEVA-TXL and BEVA/TXL were respectively the concomitant and adjuvant paclitaxel groups.

**Figure 5:**
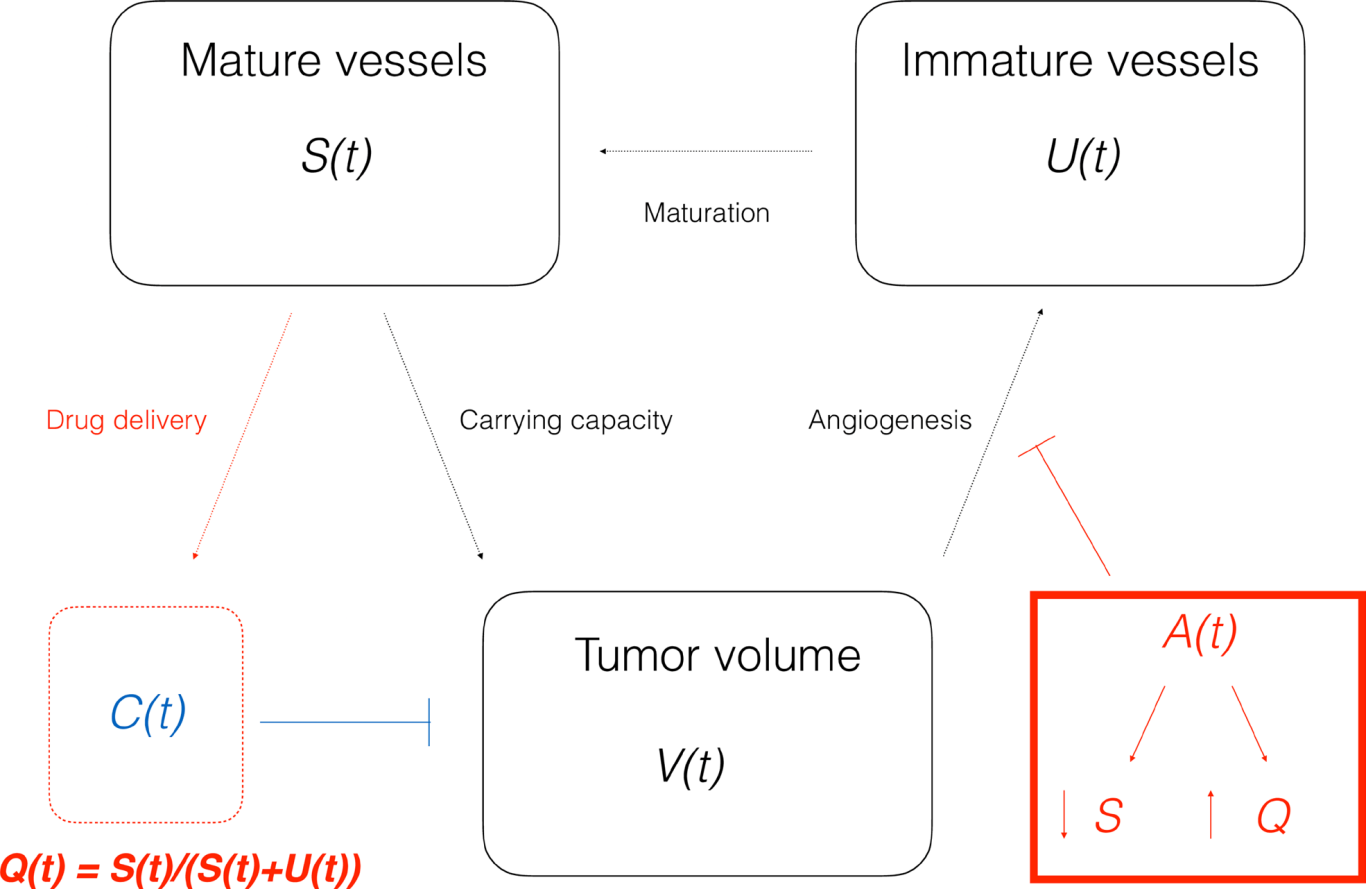
Scheme of the mathematical model (2)

The previous approach, although able to quantify the effect of vascular normalization on the drugs delivery improvement by means of $\bar{Q}$, is not suited for modeling the *dynamical changes* of the vasculature quality, which are responsible for the optimal therapeutic window in the adjuvant administration of paclitaxel. Therefore, in line with previous theoretical modeling investigations [22], we enriched the model by means of a dynamic *Q*, linked to the tumor-vasculature system in a semi-mechanistic manner. Following biological rationales [8] and previous theoretical work [18], the principle was to divide the vasculature *K(t)* into two compartments: a stable one *S(t)* (mature vessels) and an unstable one *U(t)* (immature vessels), see Figure 5. The anti-angiogenic action of the drugs was assumed to occur on *U* (rather than *S*), because it represents the component of the vasculature directly affected by neo-angiogenesis, and especially by VEGF stimulation, which is the target of bevacizumab. Then, the (macroscopic) quality of the vasculature was defined as the ratio of the stable component of the vasculature over the total amount of blood vessels. The equations write:

(2) $$\{\begin{array}{ll} \frac{dV}{dt}=aV\ln\left(\frac{S}{V}\right)-e_{TXL}SQCV & V\left(t_{I}\right)=1\\ \frac{dU}{dt}=bV-dV^{\frac{2}{3}}U-\chi U-(e_{BEVA}+5e_{CT})SQAU & U\left(t_{I}\right)=U_{0}\\ \frac{dS}{dt}=\chi U-\tau S & S\left(t_{I}\right)=S_{0}\\ \frac{dZ_{1}}{dt}=e_{TXL}SQCV-kZ_{1} & Z_{1}\left(t_{I}\right)=0\\ \frac{dZ_{2}}{dt}=kZ_{1}-kZ_{2} & Z_{2}\left(t_{I}\right)=0\\ \frac{dZ_{3}}{dt}=kZ_{2}-kZ_{3} & Z_{3\left(t_{I}\right)}=0\\ N=V+Z_{1}+Z_{2}+Z_{3} & N\left(t_{I}\right)=1\\ Q\left(t\right)=\;\frac{S(t)}{S\left(t\right)+U(t)} & \end{array}$$

### Parameters estimation

The parameters were taken the same for all the groups, the only difference being the scheduling of the therapy, and were estimated in two steps. In the first step, manual exploration of the parameter space was conducted in order to obtain visually acceptable fit between the data and the model simulation. In a second step, an optimization algorithm based on the *fminsearch* function of Matlab (version R2015a, Mathworks Inc, Nelder-Mead algorithm) was employed, following previously reported methods [44]. Data from all the groups were pooled together and the total sum of squared residuals was minimized. That is, denoting θ the vector of parameters, we minimized the following objective function:

$$J\left(\theta \right)=\;\overset{G}{\underset{g\;=\;1}{\sum }}\overset{I}{\underset{i\;=\;1}{\sum }}{\left(y_{i}^{g}-N\left(t_{i},\theta ,g\right)\right)}^{2}$$

where *g* stands for the group (scheduling) index ranging from l to *G, i* stands for the time point index ranging from l to *I*, $y_{i}^{g}$ is the data point of group *g* at time *t_i_* (mean over all the animals in the group). Due to the stiff PK profiles (Supplementary Figure 5), simulations were performed using the stiff ordinary differential equation solver *ode15s* of Matlab, with a relative tolerance of 10^−9^ to avoid numerical issues. Preliminary fit investigations constantly estimated τ to a negligible value (< 10^−10^), consistently with the observations where no size decrease was obtained. Therefore, we fixed its value to zero but nevertheless kept it in the model as it could have nonzero value in other situations. Values of the parameters resulting from the fits are reported in Supplementary Table 1.

### Cell lines

Triple negative human breast-cancer MDA-MB-231-luc-D3H2LN cells were purchased from Perkin Elmer, France. Cells were used within 6 months upon reception. This BioWare light producing cell line was derived from the MDA-231 human adenocarcinoma by stable transfection of the North American firefly gene expressed from the SV40 promoter. Upon reception, cells were gently thawed then immediately cultured per manufacturer recommendation (RPMI-1640 medium (Invitrogen) supplemented with 2 mmol/L l-glutamine (Invitrogen), 5 IU/mL penicillin/streptomycin (Eurobio), 5 IU/mL fungizon (Eurobio), and 10% of FCS (Eurobio) at 37°C in a humidified atmosphere with 5% CO2). Cells were next amplified in culture flasks, trypsinized when in exponential phase and frozen into liquid nitrogen as stock cells. The cell line was regularly authenticated on the basis of viability, recovery, growth, morphology, and bioluminescence.

### Animal experiments

All experiments were submitted and approved by the local ethical committee of the institution (#CE14, Aix Marseille Univ) and the French Ministère de L'Education Nationale, de l 'Enseignement Supérieur et de la Recherche (MENESR) prior to starting the experiments. Experiments were conducted in compliance with European regulations, based on the UKCCCR guidelines for the welfare of animals in experimental oncology [45]. Pathogen-free, immuno-compromised 6-week-old female Nod Scid γ (NSG) mice (Charles River Laboratories, France) were kept in a sterile environment for 2 weeks upon reception. Mice were maintained in sterilized filter-stopped cages kept in a sterile and thermostated cabinet throughout the experiments. They were daily monitored for signs of distress, decreased physical activity or any behavioral change. Water was supplemented with paracetamol (eq. 80 mg/kg/day) to prevent metastatic disease-related pain [46]. Animals were euthanized under anesthesia when showing signs of distress, cachexia (i.e., loss of 10% of body weight), or when tumor growth reached an apparent mass of 2 g (i.e., 2 cm3). Bodyweights were monitored twice a week as a surrogate marker for toxicity.

### Xenograft

MDA-MB-231 cells were trypsinized, counted, centrifuged (5 minutes, 1000 g) and washed twice with sterile PBS. Cells were re-suspended in RPMI-1640 with 60% of Matrigel (BD Sciences France) and maintained in ice-cooled conditions until engraftment. A volume of 50 μL containing 80 000 cells (Experiment-1) and 120 000 cells (Experiment-2) was injected in the mammary inguinal right fat pad through the nipple under gas anesthesia (2% sevofluran, (Abbott France)). A total of 84 tumor-bearing mice were required to perform the experiments. However, 90 mice were initially xenografted, to ensure that 48 (Experiment-1) + 36 mice (Experiment-2) presenting with positive and measurable tumors could be used, taking into account an estimated 5% of possible failure during the grafting procedure.

### Bioluminescence imaging

Monitoring for both primary tumor growth and metastatic spreading started one week after engraftment. Intra-peritoneal injection of firefly D-Luciferin (Perkin Elmer, 150 mg/kg) was performed in mice before starting imaging. Acquisitions started 12 minutes after Luciferine injection, the delay required to reach a plateau in bioluminescence signaling as shown in a previous study using the same mammary fat pad model [47]. For 3D bioluminescence and search for metastatic lesions, images were acquired at six different wavelengths (560 nm-660 nm) to discriminate signals depending on their depth. Acquisition and data processing were performed using IVIS Spectrum imager equipped with the Living Image 4.2 software (PerkinElmer). For Experiment-2, accuracy of the 3D imaging was compared with results from necroscopic examination at study conclusion.

### Treatments

In Experiment-1, 48 xenografted mice were divided into 6 treatment groups (*n* = 8 mice per group): control (saline injection), bevacizumab (BEVA), paclitaxel (TXL), bevacizumab + paclitaxel given concomitantly (BEVA-TXL), bevacizumab followed by paclitaxel 3 days later (BEVA/TXL), paclitaxel followed by bevacizumab 3 days later (TXL/BEVA). Treatments started 5 days after xenografting (D5). Dosing for bevacizumab and paclitaxel was 10 and 20 mg/kg respectively. A total of 3 cycles administered on a weekly basis were performed (i.e., D5, D12, D19). Based upon data collected from Experiment-1, a second set of experiment was performed, with slights changes in dosing, in sample size and inter-cure interval to increase statistical power and to maximize the differences between the groups. In Experiment-2, 36 xenografted mice were divided into 3 groups (*n* = 12 mice per group): control (saline), bevacizumab + paclitaxel given concomitantly (BEVA-TXL) and bevacizumab followed by paclitaxel 3 days later (BEVA/TXL). Treatment started 11 days after xenografting (D11). Dosing for bevacizumab and paclitaxel were 20 mg/kg for both drugs. A total of 3 cycles administered every 10 days was performed in Experiment-2 (i.e., D11, D21, D31). See the Supplementary Figure 6 for a summary of the schedules. All treatments were administered by intra-peritoneal route.

### Statistical analysis

Inter-group differences in tumor growth were tested by One-Way Anova with Tukey's HSD Post Hoc multiple comparison testing or Anova on Ranks with Dunn's method according to data distribution, using Sigma Stat 4.0. software (Systat Software, Germany). We calculated a new metastatic index defined for each group and each location as the area under the cumulative incidence divided by the duration of the experiment, thus taking into account the dynamics of the metastatic spreading (the higher the index, the more aggressive the metastatic disease). Inter-group differences in survival were analyzed by log-rank testing (R software 3.2.2, R Core Team 2013, Vienna, Austria). Standard errors on the parameters estimates were computed using previously reported methods [44, 48].

## CONCLUSIONS

In this pilot study, we have developed a simple phenomenological model that can be used to simulate the efficacy of different sequences between bevacizumab and paclitaxel, so as to determine the optimal scheduling between the drugs. This model has been voluntarily kept to a maximal simplicity so as to ensure its applicability. Although preliminary and performed on a single model, two separate mathematically-driven studies in tumor-bearing mice showed that experimental data matched model predictions, thus confirming that delaying the administration of paclitaxel after that of bevacizumab improves the efficacy of this regimen. Beyond the present issue of refining bevacizumab/paclitaxel dosing and scheduling, our model could be further customized to mimic the PK/PD relationships of antiangiogenics associated with another chemotherapeutic regimen.

## SUPPLEMENTARY MATERIALS FIGURES AND TABLES


