# Supplementary material for: Model driven optimization of antiangiogenics + cytotoxics combination: application to breast cancer mice treated with bevacizumab + paclitaxel doublet leads to reduced tumor growth and fewer metastasis
Source: Oncotarget. 2017 Feb 18;8(14):23087–98. doi: 10.18632/oncotarget.15484 (PMC5410287; doi:10.18632/oncotarget.15484)
Supplement: Supplementary file 1 [file oncotarget-08-23087-s001.pdf]

Model driven optimization of antiangiogenics + cytotoxics combination: application to breast cancer mice treated with bevacizumab + paclitaxel doublet leads to reduced tumor growth and fewer metastasis

Supplementary Materials

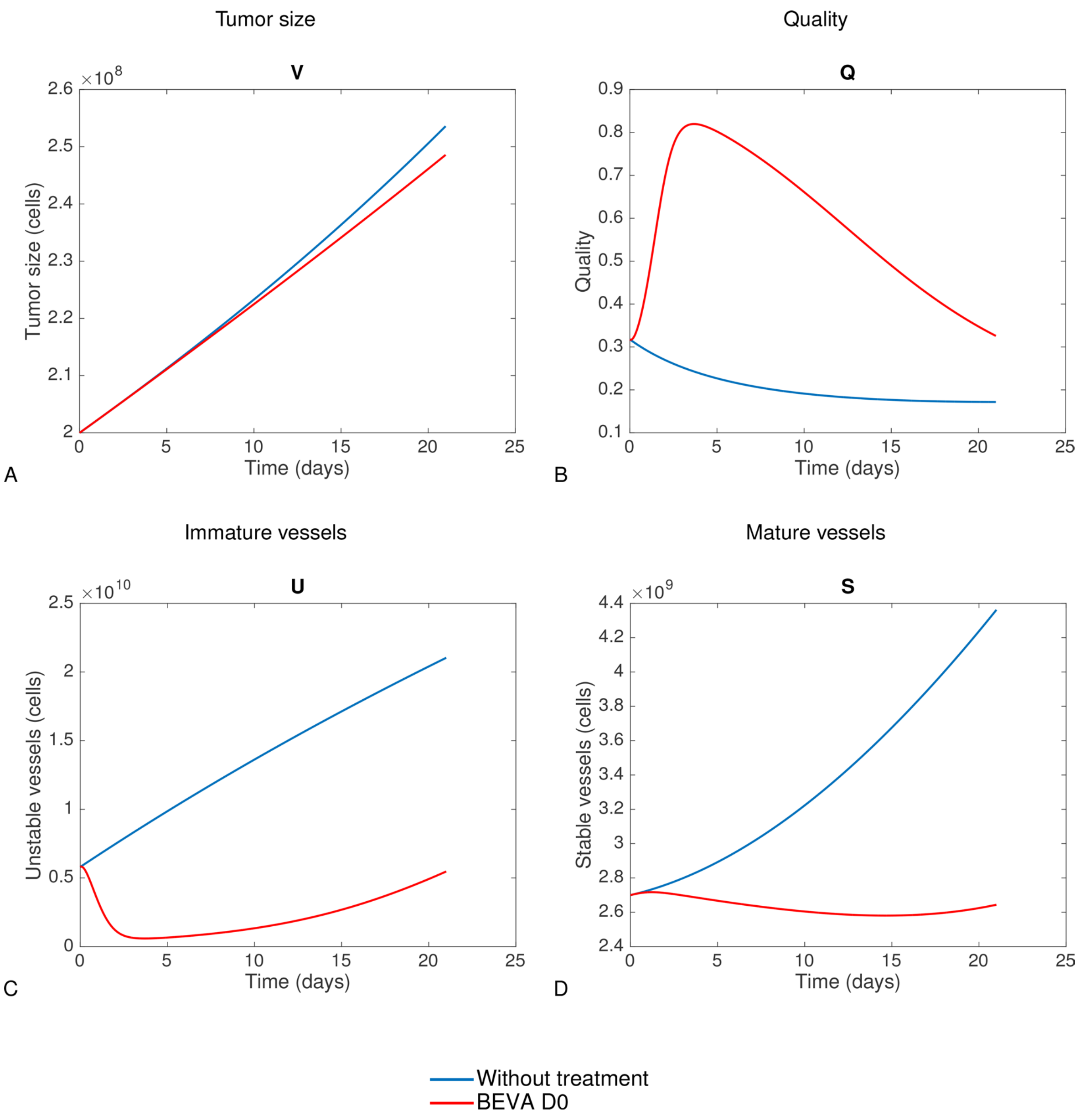

Supplementary Figure 1: Theoretical dynamics of the model (2) after a single injection of bevacizumab. Bevacizumab injection was simulated at day 0 with an initial tumor size of 2 × 108 cells and initial quality index of the vasculature of approximately 0.3. In these simulations, parameters were fixed from heuristic considerations [22].

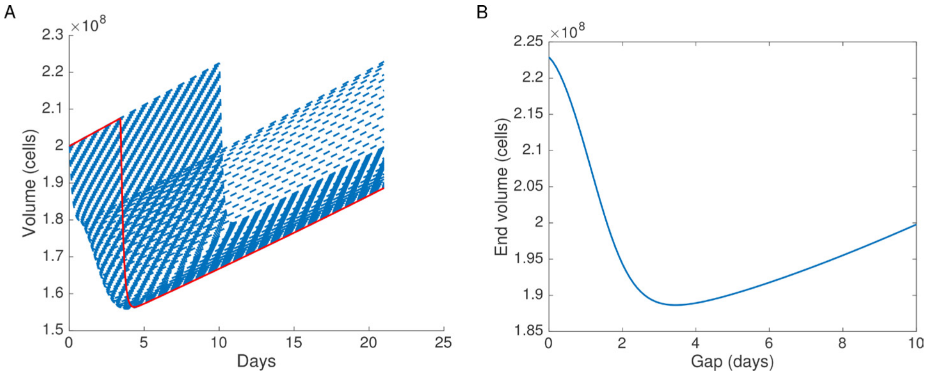

Supplementary Figure 2: Theoretical simulation of the optimal window. (**A**) Simulations of the tumor kinetics profiles after administration of bevacizumab at day 0 and administration of paclitaxel after varying time delays (gaps). Model and parameters are from [22]. (**B**) Resulting end tumor size as a function of the gap between the two drugs administrations. Minimum is reached at 3.4 days.

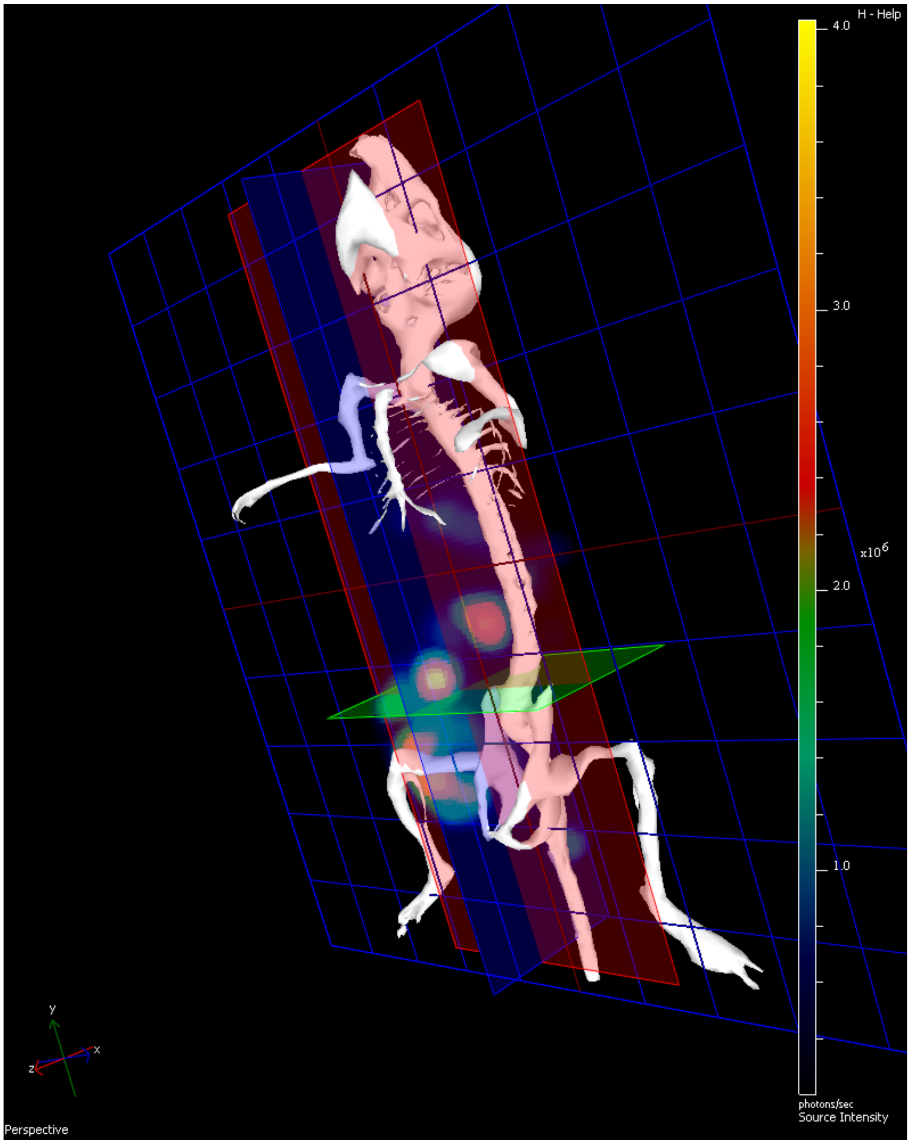

Supplementary Figure 3: Study of metastatic spreading of MDA231-Luc+ bearing mouse by 3D bioluminescence imaging after DLIT reconstruction.

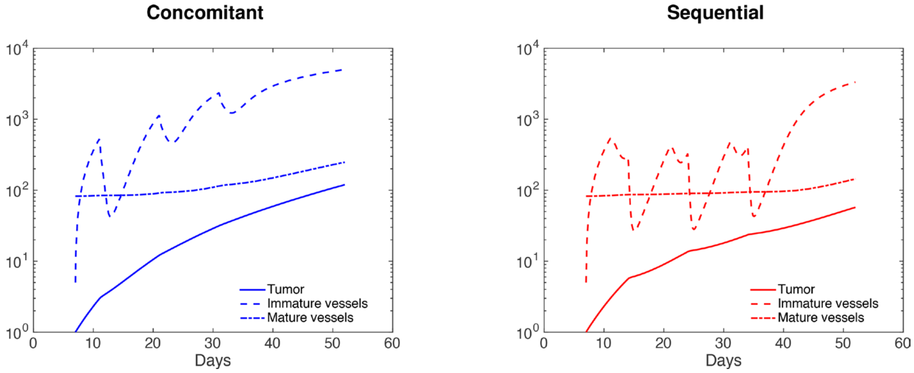

Supplementary Figure 4: Dynamics of immature and mature vasculature under the con- comitant and sequential administration schemes.

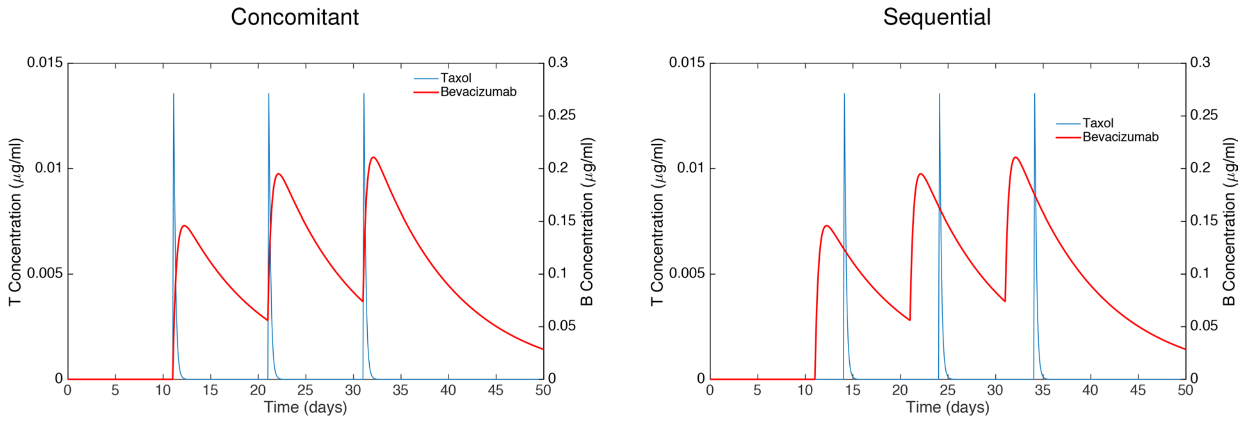

Supplementary Figure 5: Pharmacokinetics profile (Experiment-2). Pharmacokinetics (PK) profiles of bevacizumab and paclitaxel under the concomitant and sequential profiles.

PK of paclitaxel

PK of paclitaxel was retrieved from [23]. It consists in a one-compartment model with absorption that writes:

$$C(t)=\frac{D}{V}\frac{k_{01}}{k_{01}-k_{10}}\sum_{i=1}^{N} e^{-k_{10}(t-t_i)_{-e}-k_{01}(t-t_i)_{1_{t\geq ti}}}$$

where D is the administered dose of paclitaxel, V is the distribution volume, k_{01} is the absorption coefficient, k_{10} is the elimination coefficient, N is the total number of drug administrations and t_i are the paclitaxel administration times. These parameters are reported in the Supplementary Table 1. In relation to the half-life values reported in [23], the absorption and elimination coefficients are given by

$$k_{01}=\frac{\text{In}(2)}{t^{a}_{1/2}},\ k_{10}=\frac{\text{In}(2)}{t^{e}_{1/2}}$$

where $t^{\alpha}_{1/2}$ and $t^{e}_{1/2}$ are respectively the absorption and elimination half-lives of [23].

PK of bevacizumab

PK of bevacizumab was retrieved from [24]. It consisted also in a one-compartment absorption model (equation (1)) with adapted volume, absorption and elimination parameters, whose values can be found in Supplementary Table 1.

A. Expe-1

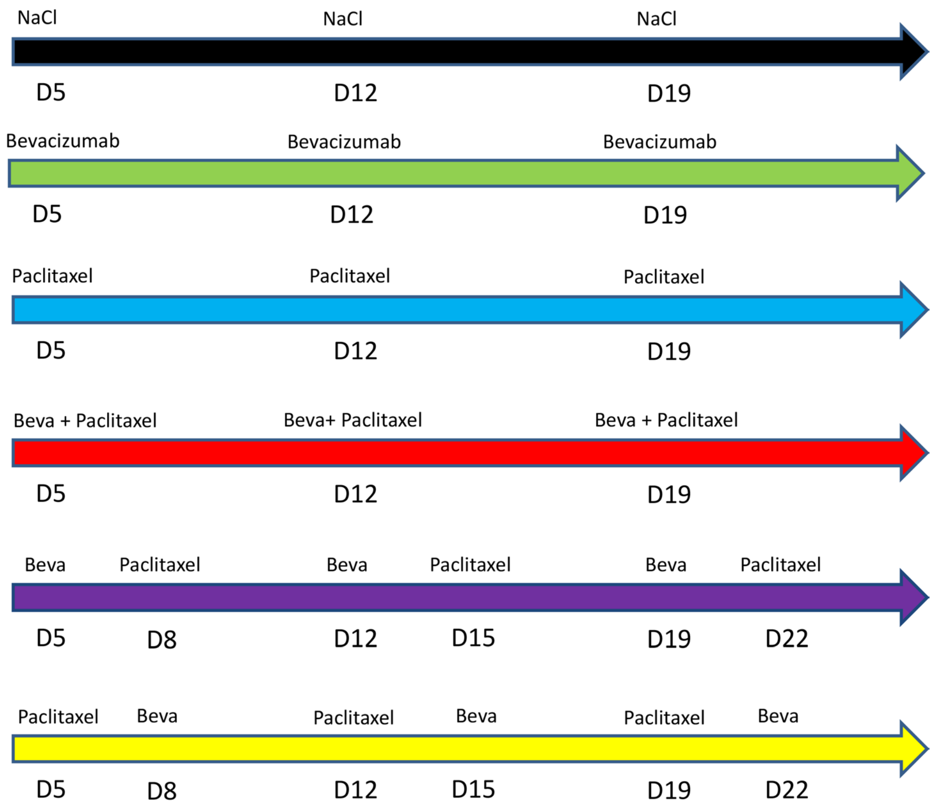

B. Expe-2

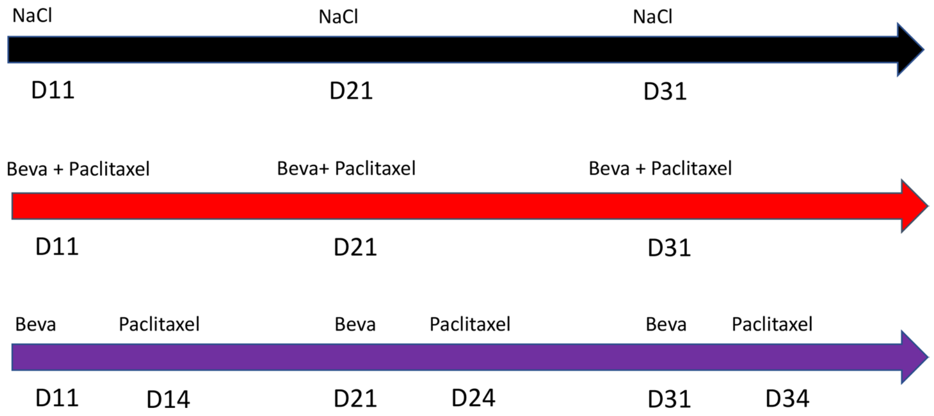

Supplementary Figure 6: Design of the schedules of the experiments.

Supplementary Table 1: Parameters estimates

	Par.	Unit	Estimate	SE (%)	Ref.
PK TXL	k_{01}	day−1	23.8	-	(22)
	k_{10}	day^{-1}	5.55	-	(22)
	V	ml	18.5	-	(22)
PK BEVA	k_{01}	day−1	2.69	-	(23)
	k_{10}	day^{-1}	0.114	-	(23)
	V	ml	2.38	-	(23)
Model (1)	a	day^{-1}	0.0786	0.0232	fit
	b	day^{-1}	1.21	0.358	fit
	d	day^{-1}	0.0202	-	(41)
	K_0	-	38.6	-	fit
	e_{TXL}	$ml \cdot mg^{-1} \cdot day^{-1}$	0.1	0.358	fit
	k	day^{-1}	0.328	51	fit
	e_{BEVA}	$ml \cdot mg^{-1} \cdot day^{-1}$	0.0812	0.0694	fit
		-	0.203	0.636	fit
Model (2)	a	day^{-1}	0.0703	0.0328	fit
	b	day^{-1}	86.8	463	fit
	d	day^{-1}	0.0745	0.508	(41)
	χ	day^{-1}	0.00203	0.0164	fit
	τ	day^{-1}	0	-	see text
	U_0	-	5	50.5	fit
	S_0	-	82.4	116	fit
	e_{TXL}	$ml \cdot mg^{-1} \cdot day^{-1}$	13.9	84.3	fit
	k	day^{-1}	8.45×10^{-9}	0.552	fit
	e_{BEVA}	$ml \cdot mg^{-1} \cdot day^{-1}$	0.494	2.73	fit

PK = pharmacokinetics. BEVA = bevacizumab. TXL = paclitaxel. ml = milliliters. biol. = biological rationale. SE = Standard Error on the parameter estimate.

Note: parameter d was converted from reference (41) into relative units. Similarly, K0 was converted from a preliminary fit to the control data set of experiment-1 and the number of injected cells converted into photons/sec.
